# The rise of non-typhoidal *Salmonella*: an emerging global public health concern

**DOI:** 10.3389/fmicb.2025.1524287

**Published:** 2025-02-04

**Authors:** Gaurav Kumar, Sandeep Kumar, Himanshu Jangid, Joydeep Dutta, Amrullah Shidiki

**Affiliations:** ^1^School of Bioengineering and Biosciences, Lovely Professional University, Phagwara, Punjab, India; ^2^Department of Microbiology, National Medical College and Teaching Hospital, Birgunj, Nepal

**Keywords:** non-typhoidal *Salmonella*, NTS, public health, salmonellosis, foodborne

## Introduction

In recent years, non-typhoidal *Salmonella* (NTS) infections have become a considerable threat to public health. This pathogen has increased in incidence and is a health concern mostly regarded as foodborne. It is highly important to appreciate the magnitude of the problem and act decisively and systematically to curb it. NTS infections are acquired mainly via food which is contaminated such as undercooked chicken, eggs, and even unpasteurized milk (Antony, 2023). An estimated 94% of salmonellosis cases result from the consumption of contaminated food (Ehuwa et al., 2021). Numerous studies have highlighted the role of non-typhoidal *Salmonella* (NTS) in foodborne outbreaks, identifying various serovars as significant contributors. For instance, *Salmonella Heidelberg* has been implicated in outbreaks (Eikmeier et al., 2018; Motladiile, 2019), alongside *S. Typhimurium* (Yang et al., 2017; Eikmeier et al., 2018) and *S. Enteritidis*, which remains a dominant serovar linked to foodborne transmission (Yang et al., 2017; Eikmeier et al., 2018; Sevilla-Navarro et al., 2020). Additionally, *S. Infantis* (Sevilla-Navarro et al., 2020), *S. Newport*, and *S. Saintpaul* (Eikmeier et al., 2018) have been recognized as causes of outbreaks across various geographical regions.

Unlike typhoidal *Salmonella* which causes typhoid fever, NTS usually cause gastroenteritis with less severe cases with symptoms such as diarrhea, vomiting and pains in the abdomen, however, it is often taken lightly (Sears et al., 2023). Most of those infected remain asymptomatic, which spreads the idea that NTS is just another case of food poisoning. On the contrary, in people whose immunity is compromised, e.g. people living with HIV or those undergoing chemotherapy, NTS can infrequently cross barriers and become deleterious. Among such individuals, moderate to severe disease may also lead to hospitalization or vegetative state and lethal outcomes in some cases. The disparity in outcomes implies that whereas recovery would be swift for a good proportion of people, some of them would have to fight for their lives.

This paper aims to provide a comprehensive overview of NTS as an emerging public health concern, focusing on its epidemiology, rising incidence rates, and research trends across diverse regions focusing on region-specific scientific contributions, the growing challenge of antimicrobial resistance, and the economic burden associated with NTS infections, highlighting the critical need for enhanced global research and public health strategies.

## Epidemiology and research trends

*Salmonella* species, and especially non-typhoidal ones (NTS), are of serious global health concern causing most cases of gastroenteritis that amount to ~93.8 million incidences and almost 155,000 deaths per annum, and the most affected region is Sub-Saharan Africa where invasive NTS death rate stands at 85.9% and specifically this region further kills under five-year-old children where in such cases the active case fatality increases to 20%. In Asia, NTS are comparatively rare, but still in Pakistan, an incidence rate of 7.2 per 100,000 people years has been reported (Table 1). The presence of risk factors like inadequate food safety measures, poor sanitation, and lack of safe drinking water explain the increased burden of NTS infections in such areas, placing additional pressure on weak health systems already (Ao et al., 2015; Das et al., 2022; Sanni et al., 2023). The two most common serovars, *Salmonella* Typhimurium and *Salmonella* Enteritidis account for the largest raft of cases globally (WHO, 2018; Balasubramanian et al., 2019; Stanaway et al., 2019; Mohakud et al., 2022; Crump et al., 2023). *Salmonella* Typhimurium is predominant in Africa and the United States (Hagedoorn et al., 2024), where it is frequently associated with beef and poultry. In contrast, *Salmonella* Enteritidis is more prevalent in Europe and Asia (Hagedoorn et al., 2024), commonly linked to imported chicken (Bloomfield et al., 2023; Mkangara, 2023). Beyond these, several other non-typhoidal *Salmonella* (NTS) serovars significantly contribute to foodborne illnesses worldwide. *Salmonella* Infantis is increasingly linked to human infections and is commonly found in chicken, pork, and retail foods in the UK (Bloomfield et al., 2023). *Salmonella* Newport, associated with beef, is a notable serovar in the U.S. with invasive disease potential. *Salmonella* Dublin, primarily tied to cattle, is prevalent in Europe and Africa but also infects humans. *Salmonella* Heidelberg is mainly found in the Americas, frequently linked to poultry. Meanwhile, Salmonella Weltevreden is prominent in seafood from Asia and North America (Hagedoorn et al., 2024).

**Table 1:** Epidemiology trends of non-typhoidal *Salmonella* (NTS) across regions.

**Region**	**Incidence**	**Common serotypes**	**Antibiotic resistance (%)**	**Mortality rate (%)**	**Hospitalization rate (%)**	**References**
Sub-Saharan Africa	535,000 cases (2017)	*S. Typhimurium* (ST313), *S. Enteritidis*	30%−50% multidrug resistance, including cephalosporins	15%−25%	High due to invasive disease	Crump et al., 2023; Stanaway et al., 2019
Southeast Asia	Varies, ~7 per 100,000 PYO (Pakistan)	*S. Typhimurium, S. Enteritidis*	Moderate resistance (especially fluoroquinolones)	< 1%	Moderate	Stanaway et al., 2019
Philippines	Moderate prevalence	*S. Enteritidis* (60.9%), *S. Typhimurium* (13%)	Ampicillin (23.2%), Ciprofloxacin (8.7%)	**Estimated 5%−10%** based on regional data	**It is likely high**, particularly in young children	Sia et al., 2023
United States	Estimated 16 per 100,000	*S. Enteritidis, S. Typhimurium*	Increasing resistance to fluoroquinolones	< 0.1%	Low	WHO, 2018
Europe	Estimated 14 per 100,000	*S. Enteritidis, S. Infantis*	Increasing multidrug resistance	Very low	Low	Hagedoorn et al., 2024

**Figure 1:**
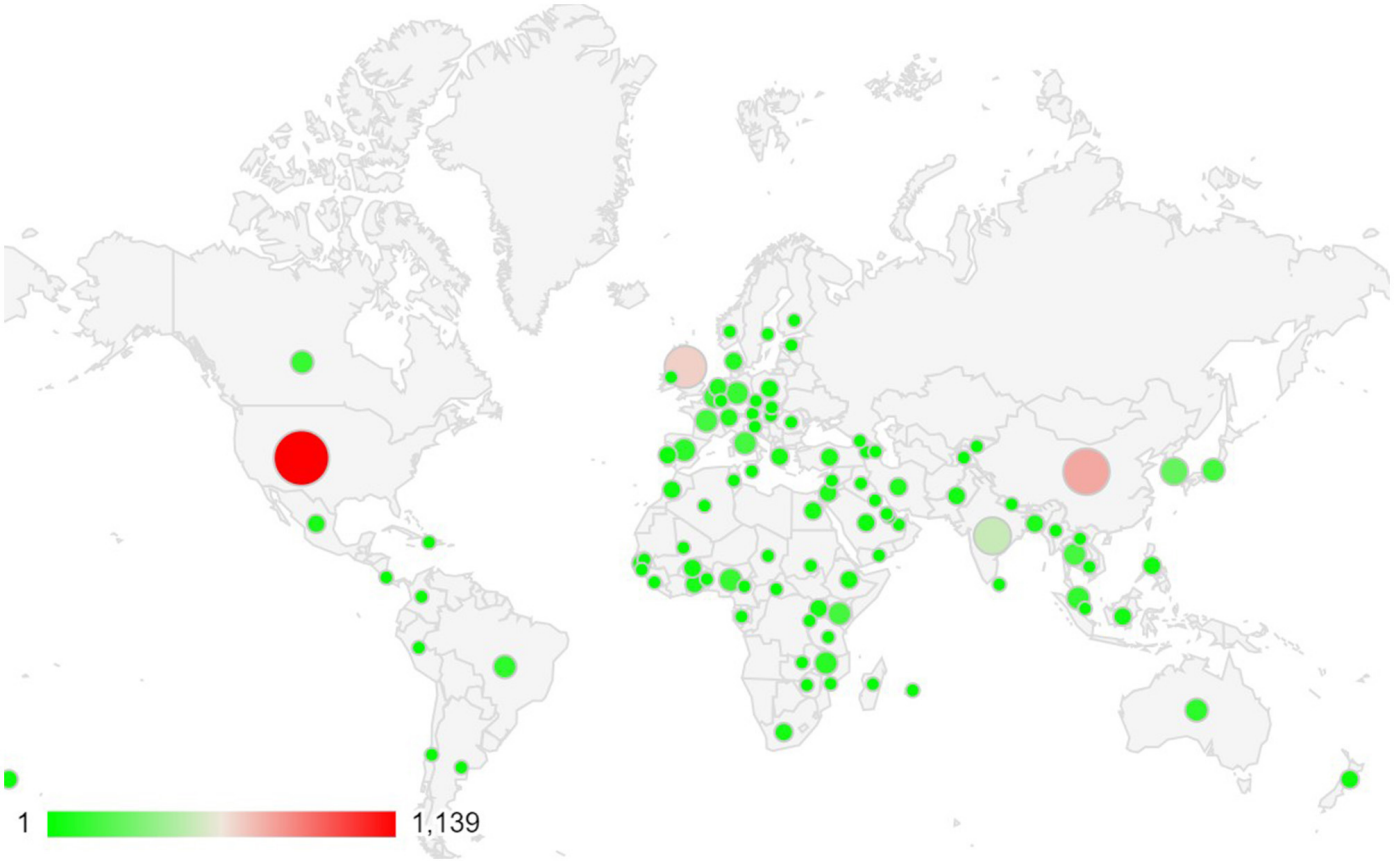
Region-specific Scientific publication frequency of scientific papers reported the NTS epidemiological. Data was collected from the Scopus Database (Between 1982-Oct 2024).

During the literature review, using the Scopus database, a bibliometric analysis of NTS research publications from 1982 to 2024 shows that most studies have been conducted in the USA, Brazil, European countries, African nations, India, China, Pakistan, and Bangladesh (Figure 1). Although this analysis highlights the geographic regions where NTS research is concentrated, it does not perfectly align with the actual epidemiological data. However, it suggests that these are the areas where NTS cases are being most actively reported and studied.

The disproportionate focus on certain regions, coupled with the lack of comprehensive data from resource-limited countries, underlines the need for more focused research in these underserved areas to better understand the disease's true impact. More targeted epidemiological studies are essential to guide public health strategies and interventions in the regions most affected by NTS.

## Rising incidences of NTS and research trends

The incidence of NTS infections has been steadily increasing, particularly affecting vulnerable groups such as young children, the elderly, and immunocompromised individuals. In these populations, NTS can escalate into severe and invasive infections, including bacteremia and meningitis, requiring prompt medical intervention (Arii et al., 2002; Magwedere et al., 2015; Ballal et al., 2016; Chen et al., 2023). However, in low-resource settings, where access to timely healthcare is often limited, the risk of life-threatening outcomes is heightened.

**Figure 2:**
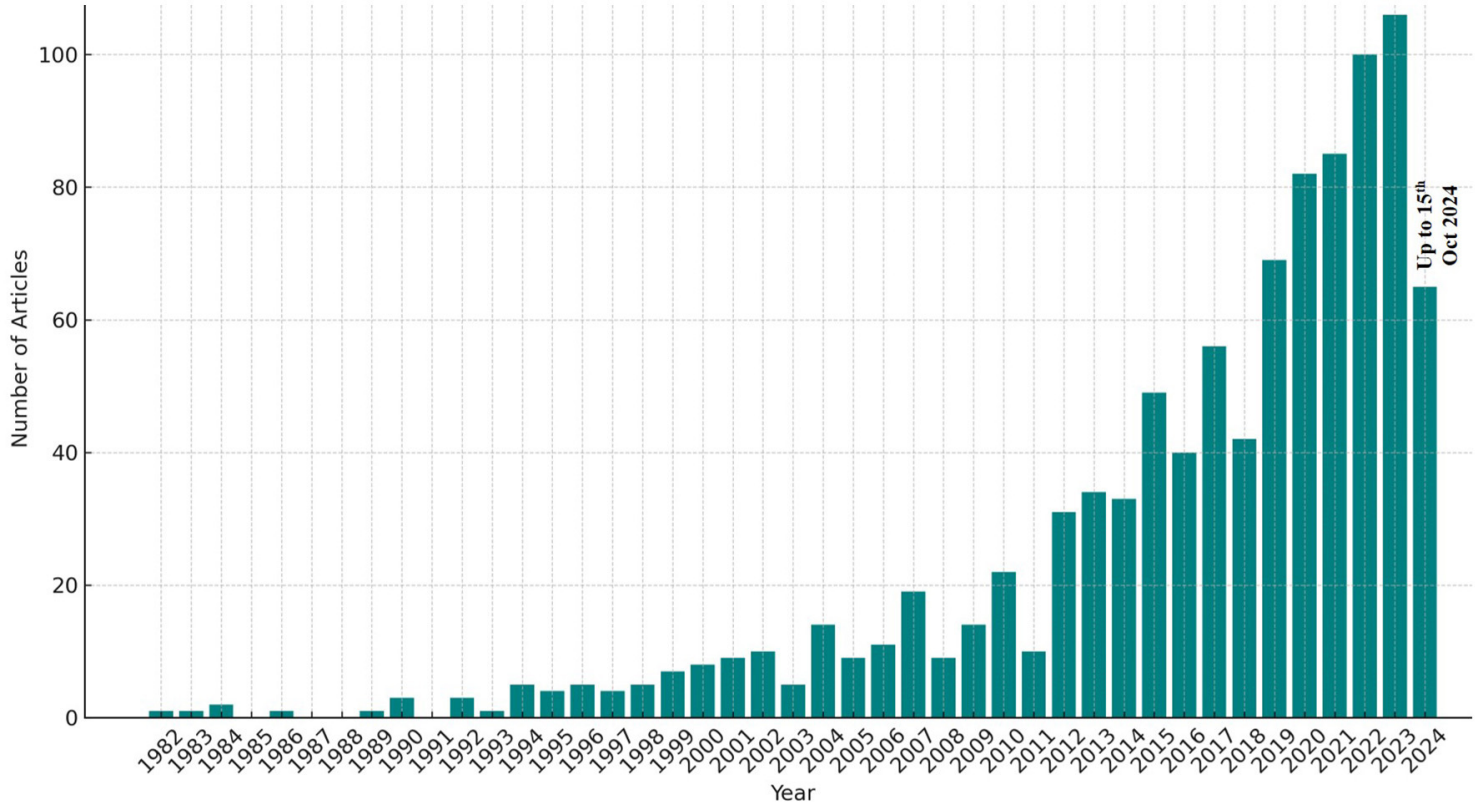
Annual publication data of scientific papers reported in the NTS epidemiological studies. Data was collected from the Scopus Database (Between 1982-Oct 2024).

As indicated by the bibliometric analysis of research publications from 1982 to 2024, there has been a noticeable rise in the number of studies focused on NTS outbreaks worldwide (Figure 2). While this increase in research publications does not directly reflect the epidemiology of NTS, it signals a growing recognition of the issue and the efforts being made to address it globally.

The rising number of NTS cases can be attributed to several factors, including inadequate food safety measures, poor hygiene, and limited public health awareness. In areas with weaker health infrastructures, the challenge of controlling NTS is more pronounced, underscoring the urgent need for comprehensive strategies to tackle this escalating public health threat.

## Drug resistance in NTS

One of the most pressing challenges with NTS is the growing occurrence of antibiotic-resistant strains. Over the years, this pathogen has become resistant to several key antibiotic classes, such as fluoroquinolones and third-generation cephalosporins, which are often used to treat severe infections. Globally, non-typhoidal *Salmonella* (NTS) shows high levels of antibiotic resistance. Among them, some regions like Guangzhou, China, documented ampicillin resistance up to 92.16%, while among Southeast Asia, which includes the Philippines, resistance to ciprofloxacin is at 8.7% (Gong et al., 2022; Sia et al., 2023). Researches indicate a sharp rise in resistance to ciprofloxacin and ceftriaxone in recent years, with some regions reporting resistance rates of up to 30% for ciprofloxacin and 25% for ceftriaxone (Hengkrawit and Tangjade, 2022; Yang et al., 2023). In countries such as Vietnam and Taiwan, multidrug resistance (MDR) among NTS strains has reached concerning levels, with studies showing that up to 53.8% of isolates were MDR between 2014 and 2019 (Hengkrawit and Tangjade, 2022). The situation is even more alarming in Bangladesh, where 94% of *Salmonella* strains from broiler chickens have been reported as multidrug-resistant (Yang et al., 2023). Multidrug resistance (MDR) has been an increasing problem with as high as 47% in China and 50% in sub-Saharan Africa (Crump et al., 2023; Gong et al., 2022). Moreover, third-generation cephalosporins, which include ceftriaxone, have also developed resistance at 2.2% level in the Philippines (Sia et al., 2023) and as high as 25% in other regions (Nelson et al., 2020; Chang et al., 2021). Sulfonamide resistance is similarly concerning, with reports showing around 43% resistance among isolates (Hengkrawit and Tangjade, 2022). A study highlighted that nearly all NTS isolates from poultry were resistant to tetracycline, emphasizing the impact of agricultural practices on resistance development (Nelson et al., 2020). Although carbapenem resistance is less common, it has been detected in some NTS strains. A study found that about 10.7% of isolates were resistant to carbapenems (Poomchuchit et al., 2021). Clearly, the resistance has reached alarming levels in view of the prevalence of antibiotics both in human healthcare and animal husbandry practices (Crump et al., 2015; Adesiji et al., 2018), resulting in the emergence of “superbugs” that are hard to treat (Wang and Sun, 2015; Siddiky et al., 2022). In countries where there is little regulation preventing people from buying antibiotics without prescriptions, the problem is even worse. There is a growing incidence of NTS strains with multi-drug resistance (Ogasawara et al., 2008; Hendriksen et al., 2019), thereby reducing the effectiveness of usual therapies and add strain to the control of Health Systems. Such resistance makes the treatment of infections limited and increases the period of illness especially for the invasive NTS. In low-resource areas where there is limited access to cheap, effective antimicrobial agents, this problem also significantly increases the death rates.

## The economic burden of NTS

NTS exerts significant economic burden in terms of healthcare expenditures, productivity losses, and the associated demand on public health systems especially in countries where the spread of infection is rampant. The combination between the cost of treatment for infections and the associated economic losses due to absenteeism is too much for any economy to bear (Mulla and Cole, 2004; Sanni et al., 2023). High-income countries also experience a similar trend, where the direct medical costs (DMCs) constitute the highest expenditure drivers for all NTS as well as invasive NTS (iNTS) cases. The average treatment price for NTS patients is USD 545.9 in Taiwan and USD 21,179.8 in Türkiye, while management and handling of iNTS differs from case to case, varying from USD 1,973.1 in Taiwan to USD 32,507.5 in the USA (Kim et al., 2024). However, it is perplexing that very few studies have been conducted on the economics in the developing and resource-limited areas (Aqeel et al., 2024), where the already strained health systems may even suffer more, thus hindering economic growth and expansion more significantly.

## Recommendations for control

The increase in NTS calls for an all-inclusive and all-embracing approach. First, there is need to say that global food safety standards must be improved. This means there should be better management of systems of food production, better hygiene practices and attitudes enforced and practices of handling food in a safe manner taught to the public (CDC Yellow Book, 2024). Maintenance of the older systems is not an option given the over-reliance on the existing systems. Instead, the governments must focus on cutting-edge detection technologies which are capable of controlling the spread of further infection in record time after outbreak. Another area that calls for attention is the management of antibiotic resistance. This entails putting measures in place to curb the use of antibiotics in food animals, encouraging the research and development of better antibiotics, and most importantly, use of antibiotics by physicians on the patient only when necessary (Fagbamila et al., 2023). In this regard, it is also important to conduct public information campaigns about the dangers of antibiotics to the public. It is also important to note that (Smith et al., 2016) improving the availability of clean water and sanitation in developing countries is important since these are public health interventions that can significantly help contain NTS as well as other enteric infections.

In addition, it is necessary to enhance global efforts for monitoring NTS outbreaks and the resistance to antibiotics. It calls for nations to work together effectively in order to determine all those populations at risk and measure the impact of the risk by activating specific measures. Organizations such as World Health Organization and Food and Agriculture Organization must lobby for better food safety and responsible use of antibiotics all over the world.

## Conclusion

The conclusion here is that there is an increasing occurrence of NTS which poses a global health threat that needs urgent and targeted measures. The completion of strengthening food safety and quality as well as addressing antibiotics resistance and ensuring proper sanitation and provision for clean water are important measures in mitigating this pathogen. The cost implications are high but such threats can be managed through strategic works and sustained commitment over a period of time in place of resources. In order to prevent millions of people's health from being compromised, such threats have to be dealt with as quickly as possible. In sickness, health groups should work toward NTS no longer being a public health threat that is under wraps.
